# Review of stochastic hybrid systems with applications in biological systems modeling and analysis

**DOI:** 10.1186/s13637-017-0061-5

**Published:** 2017-06-30

**Authors:** Xiangfang Li, Oluwaseyi Omotere, Lijun Qian, Edward R. Dougherty

**Affiliations:** 1 Department of Electrical and Computer Engineering, Prairie View A&M University, Prairie View, 77446 TX USA; 2 Department of Electrical and Computer Engineering, Texas A&M University, College Station, 77843 TX USA

**Keywords:** Stochastic hybrid systems, Biological systems modeling

## Abstract

Stochastic hybrid systems (SHS) have attracted a lot of research interests in recent years. In this paper, we review some of the recent applications of SHS to biological systems modeling and analysis. Due to the nature of molecular interactions, many biological processes can be conveniently described as a mixture of continuous and discrete phenomena employing SHS models. With the advancement of SHS theory, it is expected that insights can be obtained about biological processes such as drug effects on gene regulation. Furthermore, combining with advanced experimental methods, in silico simulations using SHS modeling techniques can be carried out for massive and rapid verification or falsification of biological hypotheses. The hope is to substitute costly and time-consuming in vitro or in vivo experiments or provide guidance for those experiments and generate better hypotheses.

## Introduction

In the last decade, one major transformation in molecular biology has been the massive scaling up of its experimental methods. Huge amounts of data on different aspects of the development and functioning of cells are generated from sequencing of the entire genome of organisms, the determination of the expression level of genes in a cell by means of DNA micro-arrays, and the identification of proteins and their interactions by high-throughput proteomic methods [1, 2].

To make good use of these data to its full potential, there is need for enhancement of experimental results with formal models of biochemical networks. An accurate and clear mathematical model that describe gene and protein interactions is essential in advancing the understanding in biology. With mathematical models, computer-based or in silico simulation and analysis of biochemical networks is possible. These in silico experiments can be used for massive and rapid verification or falsification of biological hypotheses, which can substitute in certain cases, costly and time-consuming in vitro or in vivo experiments. In addition, in silico experiments can provide guidance and predictions for in vitro and in vivo experiments, and together they can be used in a feedback arrangement [2, 3].

The possibility of combining new experimental methods, sophisticated mathematical techniques, and increasingly powerful computers has given a new lease of life to an idea as appealing as it is difficult to realize: understanding how the global behavior of an organism emerges from the interactions between components at the molecular level.

Many approaches have been proposed for modeling molecular interaction networks. One way to group the models available in the literature [4] is based on the classification of (1) continuous dynamics, for example, models that describe the evolution of concentrations of proteins, mRNAs, etc., in terms of ordinary or partial differential equations; (2) discrete dynamics, such as graph models of the interdependencies in a regulatory network, Boolean networks and their extensions, Bayesian networks, or Markov chain models. Experimental evidence suggest that neither of these classes alone is adequate for developing realistic models of molecular interaction networks [5]. Other approaches such as the interval model [6] that describing complexity and uncertainty would not be able to address hybrid discrete-continuous dynamics.

Timescale hierarchies cause biological processes to be more conveniently described as a mixture of continuous and discrete phenomena. For instance, continuous changes in chemical concentrations or the environment of a cell often trigger discrete transitions (such as the onset of mitosis or cell differentiation) that in turn influence the concentration dynamics [7]. At the level of molecular interactions, the co-occurrence of discrete and continuous dynamics is exemplified by the switch-like activation or inhibition of gene expression by regulatory proteins [8].

Hybrid discrete-continuous dynamics can play an important role in biochemical systems, realization of this fact has led to investigation on how methods developed for hybrid systems in other areas (such as air traffic management and communication networks) can be extended to biological systems [9–13]. It is fair to say that the realization of the potential of hybrid systems theory in the context of biochemical system modeling is still under investigation. In addition, the observation that many biological processes involve considerable levels of uncertainty has been gaining momentum [14, 15]. For instance, experimental observations suggest that stochastic uncertainty may play an important role in enhancing the robustness of biochemical processes [16] or may be behind the variability observed in the behavior of certain organisms [17, 18]. Stochasticity is even observed in fundamental processes such as the DNA replication itself [19, 20]. This has led researchers to attempt the development of stochastic hybrid systems (SHS) models for certain biochemical processes that aim to couple the advantages of stochastic analysis with the generality of hybrid systems.

In this review, some of the recent applications of SHS to biological and chemical systems modeling and analysis are covered. With the advancement of SHS theory, it is expected that insights can be obtained about biological processes such as drug effects on gene regulation. Furthermore, combining with advanced experimental methods, in silico simulations using SHS modeling techniques can be carried out for massive and rapid verification or falsification of biological hypotheses. The hope is to substitute costly and time-consuming in vitro or in vivo experiments or provide guidance for those experiments and generate better hypotheses.

The rest of the paper is organized as follows: In Section 2, background and basics of Stochastic Hybrid Systems (SHS) are provided. Section 3 presented the review on SHS applications to biological systems. Section 4 gives the concluding remarks.

## Brief overview of stochastic hybrid system theory

In this section, we briefly cover the basics of stochastic hybrid system theory. Many previous works on hybrid systems have focused on deterministic models. In practice, it is often desirable to introduce some levels of uncertainty in the models, this has led to the introduction of what are known as non-deterministic models in discrete event and hybrid systems.

### Hybrid systems

Hybrid systems are dynamical systems that involve the interaction of different types of dynamics. Hybrid dynamics arise out of the interaction of continuous state dynamics and discrete state dynamics [21, 22]. A state variable is called discrete if it takes on a finite (or countable) number of values and continuous if it takes values in Euclidean space $\mathbb {R}^{n}$ for some *n*≥1. By their nature, discrete states can change value only through a discrete “jump,” while continuous states can change values either through a jump or by flowing in continuous time according to a differential equation. Hybrid systems involve both these types of dynamics: discrete jumps and continuous flows [23–25]. The contribution to hybrid system mainly comes from the computer science community and the systems and control community, each coming with their own approaches. The computer science community looks at a hybrid system primarily as a discrete (computer) program interacting with an analog environment. Different reasons motivated the study of hybrid system from the systems and control community; these include hierarchical systems with a discrete decision layer and a continuous implementation layer, switching control schemes and relay control, nonlinear control systems, and discrete event systems [23, 24]. As an example, hybrid automaton model could provide a framework and terminology to discuss a range of typical features of hybrid systems [26, 27]:

#### **Definition 1**

A hybrid automaton *H* is a collection *H*=(*Q*,*X*,*I* *n* *i* *t*,*f*,*I*,*E*,*G*,*R*) where

- *Q* is a set of discrete variables and *Q* is countable;
- *X* is the continuous variables;
- *I* *n* *i* *t*⊆*Q*×*X* is a set of initial states;
- *f*:*Q*×*X*→*T* *X* is a vector field;
- *I* *n* *v*:*Q*→*P*((*X*)2^*X*^ assigns to each *q*∈*Q* an invariant set;
- *E*⊂*Q*×*Q* is a collection of discrete transitions;
- *G*:*E*→*P*((*X*) assigns to each *e*=(*q*,*q* ^′^)∈*E* a guard; and
- *R*:*E*×*X*→*P*((*X*) assigns to each *e*=(*q*,*q* ^′^)∈*E* and *x*∈*X* a reset relation.

Interested readers of hybrid systems should refer to [23–25] and proceedings of Hybrid Systems: Computation and Control (HSCC).

### Hybrid systems with randomness

The need for finer probabilistic analysis of uncertain systems has led to the study of an even wider class of hybrid systems that allow things such as random failures causing unexpected transitions from one discrete state to another, or random task execution times which affect how long the system spends in different modes [28]. For example, the events in a hybrid system may be controllable (e.g., deciding to switch gears when driving a car) or uncontrollable (e.g., some equipment failure). Uncontrollable events may occur at random points in time, in which case the hybrid system becomes stochastic. Randomness may also enter the picture through noise in one or more time-driven components of the system, in which case we must resort to stochastic differential equations [29]. In this case, if a mode switch is the result of a continuous variable reaching a certain level (e.g., a tank containing fluid whose level exceeds a specific value), then the random fashion in which this variable evolves in time affects the associated switching event.

Randomness is inherent in biochemical and biomedical systems [30, 31]. There is evidence in stochasticity or noisy process found in gene expression, introduced either through biochemical processes such as transcription and translation or fluctuations in the amounts or states of other cellular components. This stochasticity in transcriptional regulation is due partly to the random waiting times among synthesis and degradation reactions involving a finite collection of transcripts and random transitions among the discrete operator states controlling the rate of transcription [32].

The great interest of the research community in Stochastic Hybrid Systems (SHS) has produced a number of different types of stochastic hybrid models. The main difference between these classes of stochastic hybrid models lies in the way the stochasticity enters the process [33]. Some models allow diffusions to model continuous evolution [28, 34], while others do not [35, 36]. Similarly, some models force transitions to take place from certain states [28], others only allow transitions to take place at random times (e.g. using a generalized Poisson process) [34], while others allow both [36]. Examples of several types of stochastic hybrid processes are given below.

#### Piecewise deterministic Markov process

Piecewise Deterministic Markov Processes (PDMP) [36, 37] is a stochastic hybrid model with deterministic continuous dynamics in each mode, while randomness appears only in the discrete transitions. Let *Q* be a countable set of discrete states, and let ${d} : {Q}\to \mathbb {N}$ and ${X} : {Q} \to \mathbb {R}^{d(.)}$ be two maps assigning to each discrete state *i*∈*Q* a subset of $\mathbb {R}^{d(i)}$. We call the set $\mathcal {D} = \bigcup _{{i}\in {Q}}\{i\} \times {X}(i)$ the hybrid state space of the PDMP and $\alpha = ({i}, {x}) \in \mathcal {D}$ the hybrid state. The boundary of the hybrid state space is define as $\partial \mathcal {D} = \bigcup _{{i}\in {Q}}\{i\} \times \partial {X}(i)$. A vector field *f* on the hybrid state space $\mathcal {D}$ is a function ${f} : \mathcal {D} \to \mathbb {R}^{d}(.)$ assigning to each hybrid state *α*=(*i*,*x*) a direction ${f}(\alpha) \in \mathbb {R}^{d}(i)$. The flow of *f* is a function $\Phi : \mathcal {D} \times \mathbb {R} \to \mathcal {D}$ with $\Phi (\alpha, {t}) = \left [\begin {array}{l} \Phi (\alpha, {t})_{Q}\\ \Phi (\alpha, {t})_{X} \end {array}\right.$, where *Φ*(*α*,*t*)∈*Q* and *Φ*(*α*,*t*)∈*X*(*i*), such that for *α*=(*i*,*x*), *Φ*(*α*,0)=*α* and for all ${t} \in \mathbb {R}$, *Φ*_*Q*_(*α*,*t*)=*i* and $\frac {d}{dt}\Phi (\alpha, {t}) = {f}\Phi (\alpha, {t}))$. Let $\Gamma = \{\alpha \in \mathcal {D}\ \, |\, \exists (\alpha ', {t})\in \mathcal {D}\times \mathbb {R}^{+}, \alpha =\Phi (\alpha ', {t}\}; \mathcal {D}^{\ast } = \mathcal {D}\cup \Gamma $. Define $\mathcal {B}(\mathcal {D}^{\infty }) = \sigma (\bigcup _{{i}\in {Q}}\{{i}\}\times \mathcal {B}(X(i)))$ where $\mathcal {D}^{\infty } = {Q} \times \mathbb {R}^{\infty }$. The space $(\mathcal {D}^{\infty }, \mathcal {B}(\mathcal {D}^{\infty }))$ is a Borel Space and $\mathcal {B}(\mathcal {D}^{\infty })$ is a sub- *σ*-algebra of its Borel *σ*-algebra.

##### **Definition 2**

A Piecewise Deterministic Markov Process (PDMP) is a collection *H*=((*Q*,*d*,*X*),*f*,*I* *n* *i* *t*,*λ*,*R*) where

- *Q* is a countable set of discrete variables;
- ${d} : {Q} \to \mathbb {N}$ is a map giving the dimensions of the continuous state spaces;
- ${X} : {Q} \to \mathbb {R}^{d(.)}$ maps each *i*∈*Q* into a subset *X*(*i*) of $\mathbb {R}^{d(i)}$ ;
- ${f} : \mathcal {D}\to \mathbb {R}^{d(.)}$ is a vector field;
- ${Init} : \mathcal {B}(\mathcal {D}^{\infty }) \to [0,1]$ is an initial probability measure on $(\mathcal {D}^{\infty }, \mathcal {B}(\mathcal {D}^{\infty }))$, with ${Init}(\mathcal {D}^{c})$ = 0;
- $\lambda : \mathcal {D}^{\ast } \to \mathbb {R}^{+}$ is a transition rate function;
- ${R} : \mathcal {B}(\mathcal {D}^{\infty }) \times \mathcal {D}^{\ast } \to $ [0,1] is atransition measure, with ${R}(\mathcal {D}^{c},.)$ = 0.

PDMP is a non-diffusion stochastic model that finds application in optimization problems arising in resource allocation, queueing systems etc. and biochemical processes [37].

#### Switching diffusion process

Switching diffusion process (SDP) [34, 38] is a stochastic hybrid model with both continuous and discrete states.

##### **Definition 3**

A switching diffusion process (SDP) is a collection *H*=(*Q*,*d*,*X*,*f*,*I* *n* *i* *t*,*σ*,*λ*_*ij*_) where

- *Q*={1,2,...,*N*} is a finite set of discrete variables, ${N} \in \mathbb {N}$;
- ${X} = \mathbb {R}^{n}$ is the continuous state space;
- ${f} : {Q} \times {X} \to \mathbb {R}^{n}$ is a vector field;
- ${Init} : \mathcal {B}({Q} \times {X}) \to [\!0,1]$ is an initial probability measure on $({Q} \times {X}, \mathcal {B}({Q} \times {X}))$;
- $\sigma : {Q} \times {X} \to \mathbb {R}^{{n}\times {n}}$ is a state dependent real valued matrix;
- $\lambda _{ij} : {X} \to \mathbb {R}$, *i*,*j*∈*Q* are a set of *x*-dependent transition rates, with (.)≥0 if *i*≠*j* and $\sum _{{i}\in {Q}}\lambda _{{ij}}(x)$ = 0 for all *i*∈*Q*, *x*∈*X*.

SDP has the ability to depict random environments via the switching process. The continuous state evolves according to a stochastic differential equation (SDE), while the discrete state is a controlled Markov chain. The dynamics of the SDE and the transition matrix of the Markov chain depend on the hybrid state. The continuous hybrid state evolves without jumps [33].

#### Stochastic hybrid system

Stochastic hybrid systems (SHS), involve a hybrid state space, with both continuous and discrete states.

##### **Definition 4**

A stochastic hybrid system (SHS) is a collection *H*=(*Q*,*X*,*D* *o* *m*,*f*,*g*,*I* *n* *i* *t*,*G*,*R*) where

- *Q* is a countable set of discrete variables;
- ${X} = \mathbb {R}^{n}$ is the continuous state space;
- *D* *o* *m*:*Q*→2^*X*^ assigns to each *i*∈*Q* an open subset of *X*;
- ${f, g} : {Q} \times {X} \to \mathbb {R}^{n}$ are a vector field;
- ${Init} : \mathcal {B}(Q \times {X}) \to [\!0,1]$ is an initial probability measure on $({Q} \times {X}, \mathcal {B}(Q \times X))$ concentrated on $\bigcup _{i}\{i\} \times {Dom}({i}$;
- *G*:*Q*×*Q*→2^*X*^ assigns to each (*i*,*j*)∈*Q*×*Q* a guard *G*(*i*,*j*)⊂*X* such that
  - For each (*i*,*j*∈*Q*×*Q*, *G*(*i*,*j*) is a measurable subset of *∂* *D* *o* *m*(*i*) (possibly empty);
  - For each *i*∈*Q*, the family {*G*(*i*,*j*)|*j*∈*Q*} is a disjoint partition of *∂* *D* *o* *m*(*i*);
- ${K} : {Q} \times {Q} \times {X} \to \mathcal {P}$ assigns to each (*i*,*j*)∈*Q*×*Q* and *x*∈*G*(*i*,*j*) a reset probability kernel on *X* concentrated on *D* *o* *m*(*j*).

The continuous state obeys an SDE that depends on the hybrid state. Transitions occur when the continuous state hits the boundary of the state space. Whenever a transition occurs, the hybrid state is reset instantly to a new value. The value of the discrete state after the transition is determined deterministically by the hybrid state before the transition. The new value of the continuous state, on the other hand, is governed by a probability law which depends on the last hybrid state [28, 33].

## SHS applications to biological systems

In this section, sample works that employed SHS in the modeling of biological and chemical systems are reviewed. We categorized them according to their respective applications. A summary is given in Table 1.

**Table 1:** Stochastic hybrid systems applications to biological systems

Application area	Papers	Modeling mechanism	Main goal
Gene regulation	[53]	Ordinary Differential Equation and Markov Chain controlled switching	Response of a population of cancer cells to various drugs
	[54]	Ordinary Differential Equation and Markov Chain controlled switching	Subtilin Production modeling in Bacillus subtilis
	[55–57]	Ordinary Differential Equation and Markov Chain controlled switching	Parameter Identification
	[58]	Ordinary Differential Equation and Markov Chain	Lactose Regulation System of Escherichia Coli modeling
	[59]	Ordinary Differential Equation and Probabilistic Transitions	Estimation of low order statistics
DNA replication	[60]	Ordinary Differential Equation, Guarded Transition and Probabilistic Firing Time	Simulation of full genome DNA replication
	[61]	Ordinary Differential Equation, Guarded Transition and Probabilistic Firing Time	Verification of SHS
Sugar cataract development	[62, 63]	Stochastic Differential Equation, Guarded and Probabilistic Transitions	Performance of simulation techniques
	[64]	Stochastic Differential Equation, Guarded and Probabilistic Transitions	Probabilistic verification for reachability analysis
Biodiesel production system	[65]	Stochastic Differential Equation and Guarded Transition	Probabilistic verification for reachability analysis
Glycolysis	[66]	Stochastic Differential Equation, Guarded and Probabilistic Transitions	Reachability analysis of a SHS model
Population biology	[67]	Ordinary Differential Equation and Markov Processes	Moment Closure Techniques Comparison
Others	[68–77]	misc	misc

### Gene regulatory networks modeling and control

Gene regulatory network is a collection of genes that regulate the transcriptional activity of each other through their expressed proteins. The expressions of genes are intrinsically non-deterministic. One of the main reasons is the random fluctuations (noise) in the concentrations of protein species in the cell population [39]. The stochasticity in gene expressions may result from the small copy number of interacting molecular species (e.g. regulatory proteins and genes) in the relatively large cell volume [40, 41], since in a chemical system with extremely low concentrations of reacting species, a reaction occurs in a short time interval and is best viewed as a probabilistic event [42].

There are numerous models proposed for gene regulatory networks, such as those discussed in [4, 43, 44]. However, hybrid systems modeling of gene regulatory networks is still under investigation with just a few preliminary work [45–47, 53–59]

Hybrid Systems model has been applied to model interactions between GRN and periodic drug inputs [45–47, 53]. Liu et al. [47] used a nonlinear hybrid automaton to model the population dynamics of heterogenous prostate cancer cells in response to androgen suppression. The model takes into account two distinct subpopulations of prostate cancer cells: hormone sensitive cells (HSCs) and castration resistant cells (CRCs), as well as the serum androgen concentration respectively. The model has two modes: on-treatment mode and off-treatment mode. In the off-treatment mode, the androgen concentration is maintained at the normal level by homeostasis. In the on-treatment mode, the androgen is cleared at a specified rate.

Xiangfang et al. [53] proposed a model that combines cell population and genetic regulation within a single cell by using stochastic hybrid systems model that combining cell population and genetic regulation within a single cell by using SHS. They studied the response of a population of cancer cells to various drugs that targeting the proliferation and survival pathways. Their model captures both the dynamics of the cell population and the dynamics of gene regulations within each individual cell. The SHS theory was used to link the cell proliferation at the cell population level to the drug effect on the proliferation pathway at the molecular level.

**Fig. 1:**
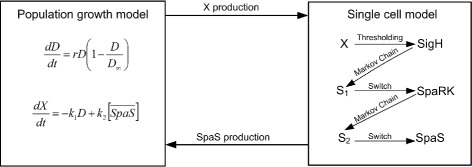
Subtilin production model

A SHS approach to analyze the dynamics of the spa genes in the subtilin production was proposed by Hu et al. [54]. Subtilin is an antibiotic produced by *Bacillus subtilis* as a regulating measure to changes in the environment, this ensure the cell benefit optimally from the available resources. The interactions between the population growth model and single-cell model of subtilin production is highlighted in Fig. 1. *D* represent the size of *B. subtilis* population, and *X* the total amount of nutrient available in the environment. The spa genes comprises of SpaR, SpaK, SpaS, SpaBTC, and SpaIFEG. The biosynthesis of subtilin is regulated by a positive feedback mechanism in which extracellular subtilin activates the two components regulatory system SpaR and SpaK that binds to a DNA motif promoting the expression of genes for subtilin biosynthesis (spaS and spaBTC) and immunity (spaIFEG). SpaR and SpaK react to form the complex SpaRK that was use in the modeling of subtilin production. SpaRK expression is controlled by a switch *S*_1_ with two states 1 (on) and 0 (off), corresponding to the cases where SigH is bound and unbound to the promoter region of the gene spaRK, respectively. The composition of SigH is turned on whenever the nutrient concentration *X* falls below a certain threshold. The switch *S*_1_ is modeled as evolving randomly at constant time interval *Δ*>0 according to a Markov chain with a probability transition matrix dependent on the concentration level of SigH. Switch *S*_2_ with two states 1 (on) and 0 (off), corresponding to the cases where activated SpaR is bound and unbound to the promoter region of the gene spaS, respectively. Moreover, *S*_2_ switches randomly at constant time interval *Δ*>0 according to a Markov chain with a probability transition matrix dependent on SpaRK. Each of *B. subtilis* cells was modeled as stochastic hybrid system. Its discrete state is (*S*_1_,*S*_2_)∈{0,1}×{0,1}, where (*S*_1_,*S*_2_)=(0,1) corresponds to the case when the switch *S*_1_ is off and the switch *S*_2_ is on, etc. So there are four possible discrete states in total. Its continuous state is ([SigH], [SpaRK], [SpaS]) $ \in \mathbb {R}^{3}$. The discrete state changes mode randomly every *Δ*>0 time according to a Markov chain with probability transition matrix and depends on the values of SigH and SpaRK at the moment of transition.

The authors in [55–57] carried out subsequent works on methods for the parameter identification of the SHS model of subtilin production by *B. subtilis* discussed above. The authors decoupled the parameter identification problem into two sub-problems, namely the estimation of the genetic network regulating subtilin production from gene expression data and the estimation of population dynamics based on nutrient and population level data, treating the switching mechanism as a Markov chain. The algorithm and methodologies they proposed efficiently identified the various parameters of interest based on both experimental data and simulation data.

**Fig. 2:**
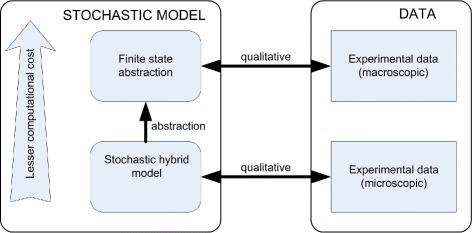
Block diagram summarizing lactose regulation system of Escherichia Coli and its finite-state abstraction

The lactose regulation system of Escherichia Coli modeling using SHS and its finite state abstraction was presented by Julius et al. [58]; this is summarized in the block diagram shown in Fig. 2. The mRNA (*M*) transcribed from the lactose operon is translated into three different gene products, among them permease (*P*) and *β*-galactosidase (*B*). Permease facilitates the influx of inducer *thio-methyl galactosidase* TMG (*T*) an external lactose from the exterior and also an opposing process, equilibrating the concentration of lactose inside the cell with the external lactose. The intrinsic noise generated by low copy numbers of molecules made SHS a suitable model for the lactose operon. The model is based on the idea that the mRNA (*M*) and the *β*-galactosidase (*B*), the two species with the lowest steady-state concentrations, were discretized and expressed as molecule counts that evolve following some Poisson processes. The other substances, internal TMG (*T*) and permease (*P*), are expressed as chemical concentrations that evolve following deterministic ODE. A finite state abstraction of the stochastic model was constructed in the form of a two-state continuous-time Markov chain, to allow for fast computation. The states of the Markov chain correspond to the low and high stable equilibria of the systems. The rates of switching between the two states are given as a function of the external TMG concentration (*T*).

Singh et al. [59] used moment closure techniques to estimate these low-order statistical moments and quantify stochastic fluctuations in different gene regulatory networks. They demonstrate that the more sophisticated form of negative feedback (using multimerization) resulting from a protein inhibiting its own transcription is more effective in suppressing noise. In addition, a two-gene cascade activation network in which the protein expressed by one gene activates another gene to express a second protein was considered. Analysis shows that the stochastic fluctuations in the population of the activated protein increases with the degree of multimerization in the activating protein.

### DNA replication

DNA replication is the process of producing duplicates of cells’ genetic information [48]. One major characteristic of eukaryotic DNA replication is its higher degree of uncertainty; hundreds to thousands of potential origins exist along the genome that fire with varying efficiencies and at different times during S-phase [49–51]

Lygeros et al. [60] developed a stochastic hybrid model that reproduces DNA replication throughout a complete genome. The need for new analytical tools to capture spatial and temporal patterns of DNA replication genome-wide becomes essential due to the large number of potential origins, coupled with system uncertainty. The various modes of DNA replication are captured by this model.

The discrete dynamics modeled the instantaneous changes in the state of the origin, *i*, this is captured by a variable, *S*_*i*_, that takes one of six values,

$$S_{i} \in \{PreR, RB, RR, LR, PostR, PassR\} $$

The origins start at the pre-replicative state (*PreR*) and end either in the passively replicated (PassR) or the post-replicating (*PostR*) states. The remaining states discriminate origins from which active forks emanate on both directions (RB), only to the left (LR) or only to the right (RR). Transitions between states are governed by “guards” (*G*), the guards are logical statements involving the variables of the model.

$$\begin{array}{@{}rcl@{}} G_{PreR\to PassR} &=& [\!X_{LN(i)} + R_{LN(i)} \ge X_{i}]  \\ &\lor&[\!X_{RN(i)} - R_{RN(i)} \le X_{i}]  \end{array} $$

$$\begin{array}{*{20}l} & G_{RB\to RR} =\ [\!X_{LN(i)} + R_{LN(i)} \ge X_{i} - L_{i}] \\ & G_{PreR\to RB} =\ [\!t \ge T_{i}] \\ & G_{RB\to LR} =\ [\!X_{RN(i)} - L_{RN(i)} \le X_{i} + R_{i}] \\ & G_{RR\to PostR} = G_{RB\to LR} \\ & G_{LR\to PostR} = G_{RB\to RR} \end{array} $$

When origin *i* fires, it gives rise to two replication forks moving away from the origin to the left and to the right. We denote by *L*_*i*_ and *R*_*i*_ the number of bases that these forks have replicated respectively. *R*_*LN*(*i*)_ and *L*_*RN*(*i*)_ denote the progress of the right and left replication forks of these neighboring origins. *L* *N*(*i*) and *R* *N*(*i*) represent origin to the left and to the right that are actively replicating at the instant under study. *X*_*i*_, *X*_*LN*(*i*)_, and *X*_*RN*(*i*)_ represents the position reached by any of the origins.

$$\begin{array}{*{20}l} LN(i) = max\{j < i| S_{j} \notin \{PreR, PostR, PassR\}\} \\ RN(i) = max\{j > i| S_{j} \notin \{PreR, PostR, PassR\}\} \end{array} $$

The continuous dynamics of the model capture evolutions that are slow compared to the discrete transitions, the only such evolution in the model is the movement of the replication forks represented by two differential equations for each active origin.

$$ \frac{{dR}_{i}(t)}{dt} = \left\{\begin{array}{ll} v(X_{i} + R_{i}(t)) & \text{if}\ S_{i}(t) \in \{RB, RR\}\\ 0 & \text{otherwise } \end{array}\right. $$

$$ \frac{{dL}_{i}(t)}{dt} = \left\{\begin{array}{ll} v(X_{i} + L_{i}(t)) & \text{if}\ S_{i}(t) \in \{RB, LR\}\\ 0 & \text{otherwise } \end{array}\right. $$

The forks move (i.e., *L*_*i*_ and *R*_*i*_ increase) at a velocity *v*(*x*) which depends on the position, *x*, of the genome currently being replicated by the fork.

The initiation events are characterized by uncertainty in time and space. Origin firing is characterized by a degree of uncertainty in both the location and time of firing of different origins, captured by the stochastic dynamics of the model. The firing time *T*_*i*_ of origin is governed by an exponential distribution.

The authors in [61] carried out subsequent works on method for the modeling and verification of SHS using the Hybrid Input Output Automaton (HIOA), a mathematical framework proposed for modeling and analysis of hybrid systems. A HIOA is an automaton that evolves discretely (transitions) and continuously (trajectories) and communicates discretely (actions) and continuously (shared variables) with its environment, e.g., other automata. This external behavior of HIOA can be used to decompose hybrid systems description and analysis, facilitating the description of complex hybrid systems. The authors built on earlier work in [60] where a model to capture the mechanics of the DNA replication process was developed in the stochastic hybrid systems framework. They provide theoretical support for these results by verifying that the proposed model using HIOA captures the mechanisms of DNA replication process by induction proofs. They showed how the SHS can be modeled using HIOA and the following being the required data for the model: (1) the length of the genome, (2) the positions of the putative origins of replication along the genome, (3) the firing times, and (4) the fork velocity.

### Other biochemical systems modeling and control

Riley et al. [62, 63] used SHS to model the biochemical process of sugar cataract development (SCD). Chemical reactions are inherently probabilistic because of the unpredictability of molecular motion; SHS is an ideal modeling tool that captures the complex dynamics of biochemical reactions. Discrete stochastic models efficiently capture the slow reactions which occur when reaction rates and concentrations are small enough. They become inefficient when there are large concentrations of molecules and/or fast reaction rates. Continuous stochastic models are ideal for modeling large concentrations of molecules and/or fast reaction rates. The continuous state *x*(*t*) evolves according to the stochastic differential equation (SDE),

$$dx = b(q,x)dt + \sigma (q,x)dw. $$

The discrete transition occurs either because the continuous state *x*(*t*) experiences a guarded transition or probabilistic transition. A guarded transition fires the instant when the guard becomes true. The firing of a probabilistic rate transition is governed by an exponential distribution characterized by the state-dependent transition rate *λ*(*q*,*x*) which is assumed to be a bounded and measurable function that is integrable for every sample path. Sugar cataract attracts water to the lens of an eye when excess of sorbitol is present, distorting light passing through the lens. Accumulation of sorbitol is affected by several factors including the amount of the enzyme sorbitol dehydrogenase (SDH). There are eight chemical species involved in the reaction involving sorbitol and SDH :*N* *A* *D* *H*(*x*_1_), *E*−*N* *A* *D* *H*(*x*_2_), *N* *A* *D* ^+^(*x*_3_), *E*−*N* *A* *D* ^+^(*x*_4_), *S* *D* *H*(*x*_5_), *F* *r* *u* *c* *t* *o* *s* *e*(*x*_6_), *S* *o* *r* *b* *i* *t* *o* *l*(*x*_7_), and the inactive form of *S* *D* *H*(*Z*). The rate of change of the concentrations of each chemical species are modeled using the SDE. Three SHS models of the biochemical process of sugar cataract development (SCD) was described. The first model describes the biochemical process of SCD. The second medicated model assumes that the effect of the drug on the system is instantaneous. The final model is designed to incorporate probabilistic delay to model absorption and metabolization. The authors showed that the probabilistically delayed medication is an ideal model for the SCD because the effect of the drug will not be immediate because of variable drug metabolism rates.

Subsequent work in [64] focused on a probabilistic verification method for computing the probability of sugar cataract formation for different chemical concentrations. The verification problem is the probability that the system execution from an arbitrary (safe) initial state will exit the safe set indicating the beginning stages of sugar cataract development. The verification method employs dynamic programming based on a discretization of the state space and therefore suffers from the curse of dimensionality.

In [65], a biodiesel production system modeled as a SHS was described and the probabilistic verification method for its reachability analysis was presented. The concentration of each of the six chemical species making up the biodiesel was modeled as a continuous variable, each of the six reactions were modeled using the SDE. The addition of more heat to the system will increase the reaction rates, the warmer the reacting chemicals can be, the faster biodiesel will be produced. However, the energy required to heat the system is a major cost of producing biodiesel, so it is important to know if a heating control system will produce biodiesel successfully under realistic conditions. The change in heating is modeled using two discrete states, which are the heating and cooling states. Between transitions, the continuous state evolves according to the corresponding SDE where the solution is understood using the It$\hat {o}$ stochastic integral. The goal of the authors was to determine the probability that the reaction will fully complete with a small excess of methanol, and to determine this, they define the set of reachable states as the set of all concentrations that satisfy required temperature for the biodiesel production. Since the system must not run out of its reactants before it runs out of methanol, the authors also define the unsafe states. The problem is to determine what is the probability that the SHS will enter the reachable set without entering the unsafe set.

The authors in [66] presented a multilevel splitting (MLS) variance reduction method for SHS. This method improves the accuracy and efficiency of Monte Carlo methods for rare events. Probabilistic analysis techniques such as Monte Carlo methods are very useful in determining reachability or safety probabilities for systems with inherent uncertainty such as SHS models. This approach was applied to reachability analysis of a SHS model of glycolysis system, a biochemical energy conversion process found in virtually every living cell. The safety probability of the system was examined, based on the defined unsafe condition for the system when the glucose drops below a certain level.

Hespanha and Singh [68, 69] presented a procedure for constructing approximate stochastic models for chemical reactions. For the number of molecules of the different species involved, one is often interested in only the first- and second-order moments, therefore, much effort can be saved by applying approximate methods to produce these low-order moments, without actually having to solve for the probability density function. A continuous state of a polynomial stochastic hybrid system (pSHS) was used to represent the population of various species involved in a chemical reaction. Polynomial stochastic hybrid systems (pSHSs) arise when the continuous vector fields in the stochastic differential equation (SDE), the reset maps, and the transition intensities are all polynomial functions of the continuous state. It was shown that for pSHSs, the dynamics of the statistical moments of its continuous states evolves according to infinite-dimensional linear ordinary differential equations (ODEs), which can be approximated by finite-dimensional nonlinear ODEs with arbitrary precision. Since the infinite-dimensional linear ODEs that describe the moment dynamics for pSHSs are still not easy to solve analytically, the finite-dimensional ODE’s provide time evolution of lower order moments for populations of species involved in a chemical reaction. Apart from providing fast simulation times and lesser computation burden compared to Monte Carlo simulations, these approximate models also open the doors to other types of analysis tools, for example, sensitivity analysis of chemical master equation. However, they provide lesser information about the probability distribution as compared to Monte Carlo simulations, for example, these approximate models do not provide information about time correlations.

A procedure for constructing approximate stochastic models for continuous-time birth-death Markov processes in population biology was proposed by Singh et al. [67]. This is done by representing the population of a species as the continuous state of a stochastic hybrid system (SHS). This SHS is characterized by reset maps that account for births and deaths, transition intensities that correspond to the birth-death rates, and trivial continuous dynamics. It has been shown that for this type of SHS, the statistical moments of the continuous state evolve according to an infinite-dimensional linear ordinary differential equation (ODE). However, for analysis purposes, it is convenient to approximate this infinite-dimensional linear ODE by a finite-dimensional nonlinear one. This procedure generally approximates some higher order moments by a nonlinear function of lower order moments, and it is called moment closure. In there work, a method for estimating lower order moments is introduced for a Markov process involving a single species, with birth and death rates being polynomials of order $s\in \mathbb {N}_{\geq 2}$. This process is modeled by a stochastic hybrid system (SHS) whose state *x* is the population of the specie. This SHS has trivial continuous dynamics $\dot {x} = 0$ and is characterized by reset maps that account for births and deaths and transition intensities that correspond to the birth-death rates.

Borowski et al. [72] modeled the spatiotemporal oscillations of the Min proteins in the bacterium *Escherichia coli* using SHS. The oscillatory system plays an important role in cell division. The stochastic model consists of a set of four interacting linear polymers, a pair of MinD and MinE polymers at either pole of the cell, each of which can be in either a growing or a shrinking state. For any fixed combination of states for the four polymers, the dynamics are deterministic and described by a system of ordinary differential equations for the lengths of the polymers. The full state of the system is thus determined by four discrete state variables (growing/shrinking) and four continuous variables (polymer lengths). Stochastic transitions between the discrete states are dependent on the cytoplasmic concentrations of the Min proteins and so indirectly on the polymer lengths. The authors then described a stochastic switching between the three possible discrete states of the state variables where the probability of switching depends on the cytosolic concentrations of MinD and MinE.

A stochastic hybrid system model is developed by Kumar et al. [71] that describes the time evolution of load position and the population counts of ants in three roles. It can be used to derive the dynamics of their statistical moments. In their model, ants switch stochastically between roles at constant, unknown probability rates, and ants in one role pull on the load with a force that acts as a proportional controller on the load velocity with unknown gain and set point. This SHS is a cascade connection of a chemical reaction network representing stochastic ant behavioral transitions followed by the deterministic dynamics of a load transported along a surface with friction. The stochastic switching of ants between behavioral states in the form of a set of chemical reactions was presented. The load dynamics was considered in the paper, they model the load as one dimensional and specified that the load is initially located at the origin and then travels only in the positive direction along the *x* axis toward the nest. The models of the ant behavioral dynamics and the load dynamics together constitute a polynomial stochastic hybrid system (pSHS).

Plotnik et al. [73] presented an approach for hybrid systems estimation that utilizes uncertain perceptional information about the system’s mode to improve tracking of its mode and continuous states. State tracking is achieved using a new form of Rao-Blackwellized particle filter called the mode-observed Gaussian Particle Filter. This new filter extends existing hybrid estimation algorithms to admit uncertain but discrete mode-related observations in addition to the information available from more traditional sensors. The framework for estimation using both traditional and perceptional information is applicable to any stochastic hybrid system with mode-related perceptional observations available. This is applicable to an automatic underwater robotic observation system that follows and films individual deep ocean animals. In order to improve the tracking of agile animals, the mode-observed Gaussian particle filter is presented to augment the measurements of relative position and water-relative velocities of the specimen with perception.

Bressloff et al. [74] consider a stochastic, conductance-based model of dendritic NMDA spikes, in which the noise originates from the stochastic opening and closing of a finite number of *N* *a* ^+^ and *N*-methyl-D-aspartate (NMDA) receptor ion channels. The resulting model takes the form of a stochastic hybrid system, in which membrane voltage evolves according to a piecewise deterministic dynamics that is coupled to a jump Markov process describing the opening and closing of the ion channels.

In [75], Bressloff et al. extended the theory of noise-induced phase synchronization to the case of a neural master equation describing the stochastic dynamics of an ensemble of uncoupled neuronal population oscillators with intrinsic and extrinsic noise. They considered simple population model that exhibits limit cycle oscillations in the deterministic limit, namely, a recurrent excitatory network with synaptic depression; inclusion of synaptic depression into the neural master equation now generates a SHS.

Farkas et al. [76] proposed a hybrid dynamical system approach to model the evolution of a pathogen that experiences different selective pressures according to a stochastic process. In every environment, the evolution of the pathogen is described by a version of the Fisher-Haldane-Wright equation while the switching between environments follows a Markov jump process. They investigated how the qualitative behavior of a simple single-host deterministic system changes when the stochastic switching process is added. They also study the stability in probability of monomorphic equilibria.

An algorithm for estimating the parameters of a given biochemical reaction network based on a stochastic hybrid model is proposed by Mikeev et al. [70]. Chemical populations in living cell can be low, therefore, ordinary differential equations (ODEs) as a modeling tool cannot be efficient, a more detailed model is necessary, which takes into account the inherently discrete and stochastic nature of chemical reactions. The authors developed efficient algorithm for the numerical approximation of the likelihood and its derivatives w.r.t. the reaction rate constants. This was an improvement to Monte Carlo sampling techniques because direct numerical solutions of stochastic hybrid systems require the solution of a system of partial differential equations. Thus, the continuous part of the state space has to be discretized, and if the discrete part of the state space is large, appropriate truncations have to be developed. They showed that for the large populations, it is often sufficient to know the expected number of molecules conditioned on the mode of the system. Therefore, the mode probabilities can be integrated over time. This allows for a fast and accurate approximation of the probability distribution of the model and can be used for the estimation of parameters based on the maximum likelihood method.

Hofbaur et al. [77] proposed an hybrid estimation scheme that can efficiently estimate complex systems with large number of modes. This was modeled using SHS that capture the large number of operational and failure modes. The authors analyzed the shortcomings of multiple model estimation schemes which track system evolutions by applying a bank of filters, one for each discrete system mode. These systems are often composed of many interconnected components that exhibit rich behaviors, due to complex, system-wide interactions.

The hybrid modeling in neuroendocrine systems is discussed in [52]. The control action of the hypothalamus is discrete (impulsive) and controls essentially continuous secretion of hormones elsewhere in the organism.

## Conclusions

The coexistence of discrete and continuous dynamics in many biological systems such as the switch-like activation or inhibition of gene expression by regulatory proteins makes hybrid systems an attractive candidate for modeling such systems. Furthermore, the uncertainties and noise in those systems demand a stochastic version of the hybrid systems. In this paper, some recent applications of stochastic hybrid systems to biological systems modeling, analysis and control are reviewed. It is envisioned that by adopting this powerful modeling and analysis approach, many biological phenomena can be correctly modeled and simulated, thus makes in silico simulations using stochastic hybrid systems modeling techniques feasible for massive and rapid verification or falsification of biological hypotheses. This may help to substitute the costly and time-consuming in vitro or in vivo experiments or at least provide guidance for those experiments and generate better hypotheses.
